# Proprioceptive Dysfunction in Focal Dystonia: From Experimental Evidence to Rehabilitation Strategies

**DOI:** 10.3389/fnhum.2014.01000

**Published:** 2014-12-09

**Authors:** Laura Avanzino, Mirta Fiorio

**Affiliations:** ^1^Section of Human Physiology, Department of Experimental Medicine, Centro Polifunzionale di Scienze Motorie, University of Genoa, Genoa, Italy; ^2^Department of Neurological and Movement Sciences, University of Verona, Verona, Italy

**Keywords:** dystonia, proprioception, sensory system, pathophysiology, rehabilitation

## Abstract

Dystonia has historically been considered a disorder of the basal ganglia, mainly affecting planning and execution of voluntary movements. This notion comes from the observation that most lesions responsible for secondary dystonia involve the basal ganglia. However, what emerges from recent research is that dystonia is linked to the dysfunction of a complex neural network that comprises basal ganglia–thalamic–frontal cortex, but also the inferior parietal cortex and the cerebellum. While dystonia is clearly a motor problem, it turned out that sensory aspects are also fundamental, especially those related to proprioception. We outline experimental evidence for proprioceptive dysfunction in focal dystonia from intrinsic sensory abnormalities to impaired sensorimotor integration, which is the process by which sensory information is used to plan and execute volitional movements. Particularly, we will focus on proprioceptive aspects of dystonia, including: (i) processing of vibratory input, (ii) temporal discrimination of two passive movements, (iii) multimodal integration of visual-tactile and proprioceptive inputs, and (iv) motor control in the absence of visual feedback. We suggest that these investigations contribute not only to a better understanding of dystonia pathophysiology, but also to develop rehabilitation strategies aimed at facilitating the processing of proprioceptive input.

Dystonia is a movement disorder characterized by sustained or intermittent muscle contractions causing abnormal, often repetitive, movements, postures, or both (Fahn, 1988). Dystonia can be classified along two axes: clinical characteristics – including body distribution – and etiology, which includes nervous system pathology and inheritance (Albanese et al., 2013). Classification by body regions identifies: focal dystonia, segmental dystonia, multifocal dystonia, generalized dystonia, and hemi-dystonia.

Focal dystonias are usually adult-onset and affect only one body region. Many cases of focal dystonia with onset in adulthood are idiopathic (meaning that dystonia is the only neurological symptom, presumably with a genetic component), though secondary, acquired cases are documented. Typical examples of focal forms are blepharospasm, oromandibular dystonia, cervical dystonia, laryngeal dystonia, and focal hand dystonia.

## Sensory Aspects of Focal Dystonia

Dystonia has been considered a “basal ganglia disorder” and attributed to a functional disturbance of the cortico-striato-thalamic-cortical circuits. This notion comes from the observation that most lesions responsible for unilateral secondary dystonia are usually confined to the putamen, caudate, globus pallidus, and thalamus (Bhatia and Marsden, 1994). The common idea is that a dysfunction of the basal ganglia and/or their connections to motor cortex plays a major role in the pathogenesis of dystonia, by influencing the final organization and execution of movement (Berardelli et al., 1998). This idea is supported by the clinical picture of dystonia, mainly characterized by motor symptoms (Currá et al., 2000; Gregori et al., 2008). However, the pathophysiology of dystonia has been largely re-discussed in the last years (Kanovský and Rosales, 2011; Avanzino and Abbruzzese, 2012; Quartarone and Hallett, 2013). Certainly, it is now widely accepted that somatosensory inputs play a substantial role in dystonia (Stamelou et al., 2012; Patel et al., 2014).

Somatosensory inputs include touch, pain, temperature, and proprioception. The contribution of the somatosensory system to the mechanism of the dystonia is supported by the following clinical aspects: (1) alleviation of dystonia with “sensory tricks” (Wissel et al., 1999; Müller et al., 2001); (2) photosensitivity and other ocular discomforts in patients with blepharospasm (Stamelou et al., 2012); (3) neck pain that often precedes cervical dystonia (Ghika et al., 1993; Stamelou et al., 2012); (4) improvement of dystonic movements after administration of local anesthetic (Kaji et al., 1995).

Apart from these symptoms and signs, patients with dystonia present mild sensory abnormalities to special testing like heat-evoked potentials (Suttrup et al., 2011) and cutaneous spatial and temporal discrimination tests (Sanger et al., 2001; Fiorio et al., 2008).

Further, in the following sections, we will focus more deeply on the neurophysiological aspects of proprioception and on the experimental evidence in support of proprioceptive dysfunction in dystonia.

## Proprioception Serves for Motor Control and Higher Order Sensation

Proprioception refers to the ability to sense the position and movements of our limbs and trunk (kinesthesia). The principal receptor involved in proprioception is the muscle spindle, which includes the primary and secondary endings of spindles. Primary endings respond to the size and speed of muscle length changes (Matthews, 1972). They are sub-served by Ia afferents and may contribute both to the sense of limb position and movement (Goodwin et al., 1972). Secondary endings do not have pronounced velocity sensitivity and signal only the length change, they are sub-served by group II afferents and may contribute to the sense of position (Matthews, 1972). After the signals from proprioceptors enter the central nervous system, a series of higher order neurons, in the cerebellum and the cerebral cortex, process the stream of proprioceptive information (for a review, see Proske and Gandevia, 2012).

It has been suggested that the input to the cerebellum is used for computations of predictive information (Wolpert et al., 1998), while that to the cerebral cortex is responsible for generating proprioceptive sensations. Neuroimaging studies showed that human kinesthesia is associated with a network of active brain areas that consists of motor areas, cerebellum, and the right fronto-parietal areas, including high-order somatosensory areas (Naito et al., 2002, 2005; Hagura et al., 2009). The neuro-anatomical correlates of kinesthesia well fit with the emerging idea that, apart from the well-established role in motor control, proprioception is largely involved in higher order functions, such as the construction of the body schema and the sense of body ownership (Proske and Gandevia, 2012).

## Experimental Evidence of Proprioceptive Dysfunction in Focal Dystonia

Among the different senses of the somatosensory system, proprioception is surely the one that is more linked to motor control. Thus, for a long time, proprioceptive dysfunction has been indicated as a good candidate for somatosensory dysfunction in dystonia.

Proprioceptive function in dystonia was studied with different approaches: muscle vibration of the arm and neck, temporal discrimination of two passive movements, reaching movements in absence of visual input, and the rubber hand illusion (RHI).

Muscle vibration is a suitable method to investigate proprioception. Vibration of the muscle belly or tendon at 50–120 Hz causes a tonic vibration reflex (TVR) that is the result of the activation of muscle spindles and γ-motoneurons. Perception of the TVR is tested by asking participants to match position and movement of the vibrated arm with the opposite arm. While the TVR *per se* is normal in different forms of focal dystonia, the perception of arm movement during the TVR is abnormal (Kaji et al., 1995; Grünewald et al., 1997; Yoneda et al., 2000). Abnormal perception occurs even for illusory movements induced by vibration. More precisely, when the vibrated arm is immobilized, an illusion of movement is produced. Since sensory information from the joints and the skin is reduced, a main contribution of Ia fibers can be suggested to account for the illusion (Rome and Grünewald, 1999; Frima and Grünewald, 2005; Frima et al., 2008). Abnormal perception of Ia afferent information with a preserved TVR suggests a central rather than a peripheral origin of the disorder. Accordingly, Bove et al. (2004) demonstrated that Ia afferent information from the neck is misinterpreted in patients with cervical dystonia (Bove et al., 2004).

A psychophysical method to investigate proprioception is the temporal discrimination of two passive movements. In this case, stimulation with needle electrodes of the first dorsal interosseus or the flexor carpi radialis muscles causes finger abduction or wrist flexion, respectively (Tinazzi et al., 2005a). Pairs of stimuli separated by short time intervals are delivered and the blindfolded subjects are asked to refer whether they perceived one or two movements (Tinazzi et al., 2005a). The temporal discrimination movement threshold is the shortest interval between two stimuli at which subjects perceived two separate movements (Tinazzi et al., 2005a). This function is preserved in patients with focal hand dystonia (Tinazzi et al., 2006a). It should be noted that this task does not necessarily require an estimation of the amount or the speed of movement, but rather the perception of the time at which the movement occurred. Hence, it could be assumed that while perception of limb velocity (sub-served by Ia afferents) is abnormal, as evidenced by the abovementioned studies on muscle vibration, perception of limb position (sub-served by group II afferents) is normal (Tinazzi et al., 2006a).

Another way to study proprioception is to ask participants to perform reaching movements with the upper limb toward a target. In the absence of visual information, this task relies on proprioception to be optimally performed. Impairments in reaching movements were shown not only in patients with dystonia of the upper limb (Inzelberg et al., 1995), but also with cervical dystonia (Pelosin et al., 2009), suggesting that the proprioceptive function can be impaired also in body parts remote from the affected district. It was hypothesized that this deficit could be due to an error in the spatial representation of the hand location or to a failure in integrating proprioceptive information with the motor output (Marinelli et al., 2011).

An original way to indirectly investigate the proprioceptive function is the RHI paradigm. The RHI is the illusion of owing an artificial hand and occurs after synchronous stroking (with paintbrushes) of the subject’s own hidden hand and a fake visible hand (Botvinick and Cohen, 1998). Typically, after synchronous stroking participants perceive their own hand as located nearer to the artificial hand – proprioceptive drift (Tsakiris and Haggard, 2005). In patients with focal hand dystonia, a dissociation was found on the affected hand between the proprioceptive drift (reduced) and the illusory feeling of ownership (preserved), whereas patients with cervical dystonia had a RHI similar to healthy subjects (Fiorio et al., 2011). The selective impairment of the proprioceptive drift in focal hand dystonia could suggest a failure in integrating the synchronous visual-tactile input with the proprioceptive location sense, because of an underlying kinesthetic deficit (Fiorio et al., 2011).

## Proprioceptive Dysfunction in Focal Dystonia: A Matter of Central Misprocessing

The abovementioned experimental evidence on proprioceptive dysfunction in focal dystonia points to an abnormality of central processing of sensory information, rather than to a peripheral problem.

Abnormal somatotopy at the cortical level was demonstrated with somatosensory-evoked potential mapping with EEG (Bara-Jimenez et al., 1998), MEG (Meunier et al., 2001), and fMRI (Butterworth et al., 2003; Nelson et al., 2009). The representations of the fingers at the cortical level are not only closer together in patients with dystonia than in healthy controls, but also in the wrong order. These abnormalities are present, and even more evident, on the unaffected side of patients with unilateral hand dystonia (Meunier et al., 2001). Interestingly, all these results in humans mimic results obtained in a primate model of dystonia, in which a plastic reorganization of the hand area in S1 was observed after months of repetitive hand movements (Byl et al., 1996). The monkeys also presented abnormal hand control and performed poorly on motor tasks, suggesting that learning-induced dedifferentiation of the sensory cortex may contribute to the genesis of dystonia.

One possible explanation for the altered cortical representation of body parts in dystonia is related to an abnormal functioning of the inhibitory interneurons that act on the sensory cortex. Tamura et al. (2008), by means of paired pulse stimulation technique, showed an impaired intracortical inhibition in S1 in focal hand dystonia. In both normal animals and humans, a conditioning (preceding) stimulus induces suppression of somatosensory-evoked potential (SEP) amplitudes evoked by a following test stimulus (Shagass and Schwartz, 1964; Angel, 1967; Wiederholt, 1978). The attenuation of the sensory evoked responses observed in healthy subjects is not observed in patients with focal hand dystonia, indicating an impaired process of “sensory gating” (Tamura et al., 2008). In addition, patients with dystonia present abnormalities in surround inhibition processes within the somatosensory system (Tinazzi et al., 2000). Surround inhibition helps to sharpen the borders of sensory afferent information, as to optimize object perception. Deficits of surround inhibition in focal dystonia were demonstrated in a SEPs study. If the median and ulnar nerves SEPs are produced together, the combined SEP should be less than the sum of the two individual ones because of mutual inhibition (Okajima et al., 1991; Huttunen et al., 1992). This is true in normal subjects, but not in patients with focal hand dystonia (Tinazzi et al., 2000).

## Link between Sensory Deficits and Motor Symptoms: Abnormal Sensorimotor Integration

Abnormal processing of somatosensory information in focal dystonia may play a crucial role in the development of motor symptoms. In this regard, it was hypothesized that in dystonia an altered somatosensory representation at the cortical level could lead to an abnormal process of sensorimotor integration in sensory, premotor, and motor cortices and in the cerebellum, which at the end results in a noisy output from the motor cortex (Konczak and Abbruzzese, 2013). In humans, sensorimotor integration can be studied at a cortical level by means of transcranial magnetic stimulation (TMS). By applying a conditioning electrical stimulus to a mixed nerve followed by a TMS stimulus on the motor cortex, inhibition of motor cortex excitability can be observed. These effects, more evident at interstimulus intervals of 20 and 200 ms, are described as short-latency (SAI) and long-latency (LAI) afferent inhibition, respectively (Tokimura et al., 2000). For SAI, it is not clear yet if the effect is mediated directly through somatosensory projections to the primary motor cortex (M1) or indirectly through S1. LAI probably involves other pathways, such as the basal ganglia or cortical association areas. LAI is defective in patients with focal hand dystonia (Abbruzzese et al., 2001), while SAI is normal (Avanzino et al., 2008), indicating abnormal central processing of sensory inputs. Another way of studying sensorimotor integration is to combine TMS with low amplitude muscle vibration. If the TMS pulse is delivered over M1 after 1 s of hand muscle vibration, M1 excitability is increased in the vibrated muscle and decreased in adjacent muscles (Rosenkranz and Rothwell, 2003). Further, the activity of the inhibitory interneurons targeting the vibrated muscle is reduced, and the opposite changes occur in surrounding muscles (Rosenkranz and Rothwell, 2003). This pattern of sensorimotor interaction is abnormal in patients with focal hand dystonia, with a little effect of vibration on cortical excitability (Rosenkranz et al., 2005).

In this scenario, it was also hypothesized an involvement of the cerebellum (Avanzino and Abbruzzese, 2012). It is well established that the cerebellum plays a primary role in predictive (feed-forward) motor control (Bastian, 2006). In the “forward” model, current body state and motor commands are combined to estimate body state in the future (Miall et al., 1993; Wolpert et al., 1995; Paulin, 2005). In this model, proprioception is the main source of information that the cerebellum processes in order to depict the current sensory state. Recent studies showed alteration in forward model prediction of sensory outcome of self produced (Lee et al., 2013) and observed (Avanzino et al., 2013) motor actions in patients with focal dystonia. Particularly, it was shown that patients with focal hand dystonia presented an abnormal performance on the temporal expectation of visually perceived handwriting movements, likely due to an abnormality in the integrative role of the cerebellum over sensory and motor cortical areas (Avanzino et al., 2013). This hypothesis finds support in the modern view of dystonia pathophysiology, which suggests that focal dystonia is linked to the dysfunction of a complex neural network comprising not only the basal ganglia–thalamic–frontal cortex circuit, but also the inferior parietal cortex and the cerebellum (Poston and Eidelberg, 2012; Hutchinson et al., 2014).

Finally, neuromodulation studies supported the idea that abnormal premotor–motor interactions may also play a role in the pathophysiology of focal dystonia (Murase et al., 2005; Huang et al., 2012; Furuya et al., 2014).

Hence, an aberrant activity in every node of the sensorimotor network (the sensory cortex, the premotor–motor cortex, and cerebellum) may play a role in inducing dystonic symptoms.

## Rehabilitation Strategies Based on Proprioception

**Figure 1:**
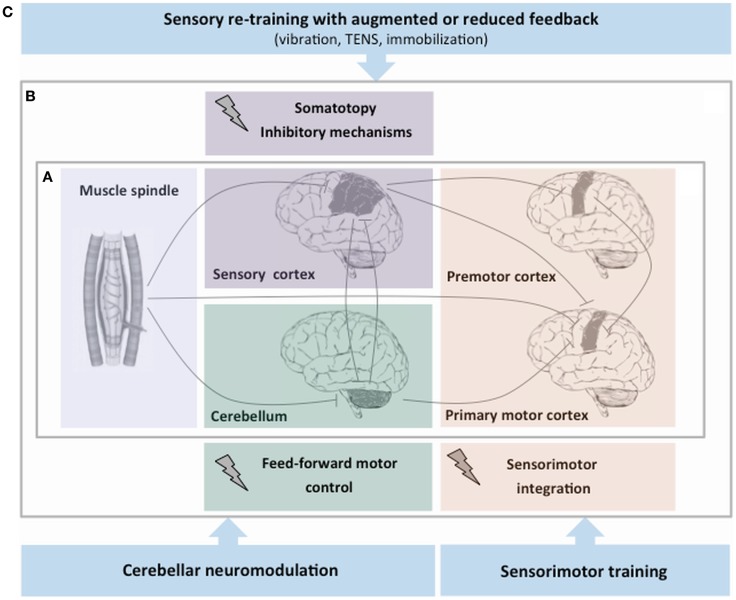
**Simplified schema of proprioceptive dysfunction and rehabilitation strategies aimed at facilitating the proprioceptive processing in focal dystonia**. **(A)** After the proprioceptive signals from muscle spindles enter the central nervous system, a series of higher order neurons located in the cortex and in subcortical structures process this information. **(B)** Experimental evidence on proprioceptive dysfunction in focal dystonia points to an abnormality of central processing of sensory information at different levels of the central nervous system: the sensory cortex (abnormal somatotopy and inhibitory mechanisms), the premotor–motor cortex (malfunctioning sensorimotor integration process), and the cerebellum (altered feed-forward motor control). **(C)** The hypothesis that dystonia could be primarily a sensorimotor disorder has led the suggestion that rehabilitation strategies may target the abnormal sensory processing of proprioceptive information.

The hypothesis that focal dystonia could be a sensorimotor disorder lead to the suggestion that rehabilitation strategies aimed at facilitating the processing of proprioceptive input could be beneficial (Figure 1). These approaches modulate sensory processing by means of sensory retraining and learning-based sensorimotor re-education.

There is emerging evidence that vibration induces sensory reorganization at a central level (Avanzino et al., 2014) and may help to reduce involuntary muscle activity. Rosenkranz et al. (2008) adopted a proprioceptive training consisting in the vibration of the abductor pollicis brevis muscle at a frequency of 80 Hz for 15 min. This procedure reversed the abnormal sensorimotor organization of the hand area in patients with focal dystonia (Rosenkranz et al., 2008). Most importantly, this intervention had a beneficial impact on the patients’ hand motor functions (Rosenkranz et al., 2009). It is important to mention that also in a single case of cervical dystonia long-term neck muscle vibration was associated with improvements in head and trunk position (Karnath et al., 2000).

Also transcutaneous electrical nerve stimulation (TENS) and kinesio-taping were used for sensory retraining. Improvement of dystonic symptoms in patients with focal hand dystonia was observed after 2 weeks of TENS of the forearm flexor muscle and lasted for 3 weeks after intervention (Tinazzi et al., 2005b, 2006b). Likely, TENS re-established a balanced activation between agonist and antagonist muscles (Tinazzi et al., 2005b). In a recent pilot study, kinesio-taping was used as a means of inducing muscle-stretching and promoting better sensory processing in patients with focal hand and cervical dystonia (Pelosin et al., 2013).

An opposite approach is sensory deprivation by means of immobilization. In patients with focal hand dystonia, immobilization of the upper limb with orthesis re-established the cortical map topography (Lissek et al., 2009; Roll et al., 2012). Selective immobilization can be applied together with motor training (Candia et al., 2005; Zeuner et al., 2005). A study in 10 patients with focal hand dystonia applied motor exercise of one finger while the other four were immobilized by a splint, for a period of 4–12 weeks (Zeuner et al., 2005). A highly variable subjective improvement, assessed by a self-rating scale, was observed.

Learning-based sensorimotor re-education can be achieved in cervical dystonia with visual or auditory EMG biofeedback techniques (Cleeland, 1973; Korein et al., 1976; Leplow, 1990). The underlying principle is to gain more volitional control over the abnormally active muscles. In patients with focal hand dystonia, instead, sensorimotor re-education has been based on a relearning process where the goal is to learn a new way of writing. In a relatively large and controlled study of 50 patients, Schenk et al. (2004) found an improvement of various writing performance components by applying individually tailored writing exercises one session per week for 4 months.

Finally, a recent study exploited neuromodulation in cervical dystonia with the aim of targeting the abnormal cerebellar function (Koch et al., 2014). Cerebellar continuous theta burst stimulation for 2 weeks induced a small but significant clinical improvement and a modification of the connectivity between the cerebellum and M1, suggesting that the cerebello-thalamo-cortical circuit could be a potential target to partially reduce some dystonic symptoms and deserves further in-depth studies.

## Concluding Remarks

The study of proprioceptive function in focal dystonia could help not only to clarify the pathophysiology, but also to highlight new rehabilitation strategies that positively impact on the motor symptoms.

## Author Contributions

Both Laura Avanzino and Mirta Fiorio contributed to the conception, drafting, and revision of the work. Further, Laura Avanzino and Mirta Fiorio made the final approval of the version to be published.

## Conflict of Interest Statement

The authors declare that the research was conducted in the absence of any commercial or financial relationships that could be construed as a potential conflict of interest.
